# 

**Published:** 1915-12

**Authors:** 


### Local Medical Notes.

**University of Bristol.**—Students of the University have recently passed the following examinations :—

UNIVERSITY OF LONDON.—*M.B., Ch.B.—Part II.:* R. B. Britton ; *Anatomy and Physiology:* N. Kemm.

CONJOINT EXAMINING BOARD OF ENGLAND.—*Medicine, Surgery, Midwifery:* V. M. Coates,* C. L. Emmerson,* R. Heaton, B.A.,* H. N. Morgan,* and A. P. Deverell.* *Medicine:* A. W. Adams. *Surgery:* D. M. Smith.

L.D.S., R.C.S. ENG.—W. S. Hazell,* C. Murray Sheriff,* W. H. B. Stride,* R. A. Waters,* A. E. Young,* A. J. Constance.

UNIVERSITY OF BRISTOL.—*M.B., Ch.B.—Final Examination, Part I.:* Hilda K. Ewens ; *2nd Examination, Part II.:* D. G. Cossham ; *1st Examination:* Hilda M. Brown, J. R. Duerden, A. J. Keivill, T. H. A. Pinniger, Marjorie S. Neville. *B.D.S., 1st Examination:* Doris B. Milton (in Zoology completing examination), G. N. O'Season. *Diploma in Dental Surgery—1st Examination:* J. L. Delicati, C. F. Long, W. H. Shipway. *Diploma in Public Health:* A. Semple, R. E. Thomas.

APPOINTMENTS.

Charles Ferrier Walters, F.R.C.S. Eng., L.R.C.P. Lond., has been appointed Honorary Surgeon to the Bristol Royal Infirmary.

Hubert Chitty, M.B., M.S. Lond., F.R.C.S. Eng., L.R.C.P. Lond., has been appointed Honorary Assistant Surgeon to the Bristol Royal Infirmary.

Capt. J. P. I. Harty has been appointed Specialist in Ear, Nose and Throat Affections at No. 6 General Hospital, Rouen.

---

**Despatches.**—The following local medical men have recently been mentioned in Despatches (October 15th) :—Thurston, H. C., Col., R.A.M.C., C.M.G. ; Green-Armytage, V. B., Capt., I.M.S. ; Quinlan, W. T., Capt., R.A.M.C. (S.R.) ; Mumford, W. G., M.B., F.R.S., temporary Capt., R.A.M.C. ; Costobadie, H. P., F.R.C.S., temporary Lieut., R.A.M.C. ; Young, J., M.D., Lt.-Col., R.A.M.C.(T.), 3rd S.M.F.A. ; Williamson, G. S., Capt., R.A.M.C.(T.), 3rd S.M.F.A. ; Smythe, H. J. D., Capt., R.A.M.C.(T.), 3rd S.M.F.A. ; Daukes, S. H., M.B., Capt., R.A.M.C.(T.), Lond. Sanit. Co.

Wookey, E. E., Lieut., 4th Glos. (T.F.).

* Denotes qualification.

### INDEX.

Almond, Dr. G. H. H.—The clinical examination of stools, 95.
Antisepsis in wounds, Sepsis and, 206.
Antityphoid inoculations, Value of, 65.
Army in the field, Women doctors with the—Dr. Helen B. Hanson, 88.
Asphyxiating gases, 113.

B. coli infections in infancy and childhood, 61.
Baron, Dr. Barclay J., appointed Lord Mayor, 218.
Bleeding, Uses of, 71.
Bones, Sarcoma of the long, 58.
**Books, Reviews of**—
Anatomy, Practical—D. J. Cunningham, 117.
Bainbridge and J. A. Menzies, Drs. F. A. —Essentials of physiology, 119.
Bayliss, W.—Principles of general physiology, 215.
Billings, John Shaw: a memoir—Dr. F. H. Garrison, 211.
Biology of the blood-cells, The—Dr. O. C Gruner, 122.
Blood-cells, The biology of the—Dr. O. C. Gruner, 122.
Brown and J. K. Murphy, Dr. W. L. [Eds.]—The practitioner's encyclopædia of medical treatment, 213.
Calot, F.—Indispensable orthopædics, 209.
Cancer problem, The—C. E. Green, 214.
Catalogue (Index-) of the library of the Surgeon-General's office, 217.
Child's diet, The—J. S. Curgenven, 122.
Consumptives, After-treatment of—Dr. H. Vallow, 115.
Cunningham's manual of practical anatomy, 117.
Curgenven, J. S.—The child's diet, 122.
Despard, L. L.—Text-book of massage, 123.
Dupuy, G. M.—The stretcher bearer, 216.
Encyclopedia medica, 117, 216.
Encyclopædia of medical treatment—Drs. W. L. Brown and J. K. Murphy [Eds.] 213.
Fitzwilliams, Dr. D. C. L.—A nursing manual, 215.
Fractures, Operative treatment of—Sir W. A. Lane, 120.
Garrison, Dr. F. H.—John Shaw Billings: a memoir, 211.
Gibson, Dr. A. G.—A hand-book for the *post-mortem* room, 64.

**Books, Reviews of** (*continued*)—
Glaister, Dr. J.—Text-book of medical jurisprudence and toxicology, 114.
Gould, Sir A. P.—Elements of surgical diagnosis, 120.
Green, C. E.—The cancer problem, 214.
Gruner, Dr. O. C.—The biology of the blood-cells, 122.
Heart in early life, The—Dr. G. A. Sutherland, 115.
Hewat, Dr. A. F.—The examination of the urine, 121.
Husband, Dr. H. A.—The students' pocket prescriber, 121.
Hutchison and J. Sherren, Dr. R. [Eds.]—An index of treatment, 213.
Kelson, W. H.—Diseases of the throat nose and ear, 216.
Kenwood, Dr. H. R.—Public health laboratory work, 117.
Lane, Sir W. A.—The operative treatment of fractures, 120.
Lejars, F.—Urgent surgery, 116.
Life: a poem—Dr. B. G. Morison, 214.
Life, On the means for the prolongation of —Sir H. Weber, 121.
MacMunn, Dr. C. A.—Spectrum analysis, 64.
Manson, Sir P.—Tropical diseases, 217.
Massage, Text-book of—L. L. Despard, 123.
Mechano - therapeutics — Dr. G. de Swietochowski, 123.
Medical jurisprudence, Text-book of—Dr. J. Glaister, 114.
Medical treatment, The practitioner's encyclopædia of—Dr. W. L. Brown and J. K. Murphy [Eds.], 213.
Menzies, Drs. F. A. Bainbridge and J. A.—Essentials of physiology 119.
Michels, R.—Sexual ethics, 63.
Millard, Dr. C. K.—The vaccination question in the light of modern experience, 116.
Morison, Dr. B. G.—Life · a poem, 214.
Murphy, Dr. W. L. Brown and J. K. [Eds.]—The practitioner's encyclopædia of medical treatment, 213.
Nursing manual—Dr. D. C. L. Fitzwilliams, 215.
Orthopædics, Indispensable—F. Calot, 209.
Paton, Dr. D. N.—Essentials of human physiology, 119.
Photographic exposure record and diary, 122.
Physiology, Essentials of—Drs. F. A. Bainbridge and J. A. Menzies, 119.
Physiology, Essentials of human—Dr. D. N. Paton, 119.
Physiology, General—W. Bayliss, 215.
Physiology in surgical and general practice, The newer—Dr. A. R. Short, 118.
*Post-mortem* handbook—Dr. A. G. Gibson, 64.

**Books, Reviews of** (*continued*)—
Prescriber, Pocket—Dr. H. A. Husband, 121.
Public health laboratory work—Dr. H. R. Kenwood, 117.
Sexual ethics—R. Michels, 63.
Sherren, Dr. R. Hutchison and J. [Eds.] —An index of treatment, 213.
Shipley, Dr. A. E.—The minor horrors of war, 123.
Short, Dr. A. R.—The newer physiology in surgical and general practice, 118.
Spectrum analysis—Dr. C. A. MacMunn, 64.
Stretcher bearer, The—G. M. Dupuy, 216.
Surgical diagnosis—Sir A. P. Gould, 120.
Surgery, Urgent—F. Lejars, 116.
Sutherland, Dr. G. A.—The heart in early life, 115.
de Swietochowski, Dr. G.—Mechano-therapeutics in general practice, 123.
Thomson, Dr. J. A.—Outlines of zoology, 118.
Throat, nose and ear, Diseases of—Dr. W. H. Kelson, 216.
Treatment, An index of—Dr. R. Hutchison and J. Sherren [Eds.], 213.
Tropical diseases—Sir P. Manson, 217.
Urine, Examination of the—Dr. A. F. Hewat, 121.
Vaccination question in the light of modern experience—Dr. C. K. Millard, 116.
Vallow, Dr. H.—The inevitable complement—the care and after-treatment of consumptives, 115.
War, The minor horrors of—Dr. A. E. Shipley, 123.
Weber, Sir H.—On means for the prolongation of life, 121.
Zoology, Outlines of—Dr. J. A. Thomson, 118.
Bristol Medico-Chirurgical Society, 71, 226.
Bristol Medico-Chirurgical Society, The foundation of the—Dr. G. Parker, 129.
British Medical Association, Notes on representative meeting of, 220.
Bullets, Removal of, and other metallic foreign bodies — Dr. R. G. P. Lansdown, 157.

Camphor, Pharmacology of, 68.
Cardiac diseases and disorders in warfare—Dr. C. Coombs, 149.
Cerebro-spinal fluid, Colloidal gold reaction in, 54.
Charles, Dr. J. R.—Report on medicine, 54.
Children, Diseases of, Report on—Dr. E. C. Williams, 61.
Clarke, Dr. J. M.—On toxic polyneuritis of the motor type, 9.
Coombs, Dr. C.—Cardiac diseases and disorders in warfare, 149.
Cotton-seed dermatitis and its cause, pediculoides ventricosus—Dr. J. A. Nixon, 73.
Cross, F. R.—The Long Fox lecture, 163.

Davies, N.—Neuritis : its definition and successful treatment, 81.
Dermatitis, Cotton-seed, and its cause, pediculoides ventricosus—Dr. J. A. Nixon, 73.
Despatches, Local medical men mentioned in, 231.
Drainage continu par le gaz oxygène —Dr. Renoirdre, 39.
Dysentery due to Y bacillus—R. H. Norgate and Dr. I. W. Hall, 44.

Eager, Dr. R., Obituary notice of, 228.
Editorial notes, 65, 218.
Epilepsy in later life, 71.
Evolution of the sense of sight—F. R. Cross, 163.
Eyes, Gunshot injuries to the—E. H. E. Stack, 198.

Fox, Dr. C. H., Obituary notice of, 229.
Fox (Long) lecture, The—F. R. Cross, 163.
France, Surgical experiences in, 72.
Frontal sinus suppuration, The pernasal operation for—Dr. P. Watson-Williams, 24.

Gastro-enteric functions, Sympathetic nervous system and the, 55.
Gilbert, Lieut. J. W., Military award, 219.
Gold reaction in cerebro-spinal fluid, 54.
Goodden, Lt. H. W., Obituary notice of, 123.
Gunshot injuries to the eyes—E. H. E. Stack, 198.

Hall, R. H. Norgate and Dr. I. W.—An outbreak of institutional dysentery due to the Y bacillus, 44.
Hanson, Dr. Helen B.—Women doctors with the army in the field, 88.

Insurance, Sickness, for medical men, 225.
Intestinal stasis—Sir W. A. Lane, 1.

Lane, Sir W. A.—Intestinal stasis, 1.
Lansdown, Dr. R. G. P.—Removal of bullets and other metallic foreign bodies, 157.
Library of the Bristol Medico-Chirurgical Society, 226.
Local medical notes, 72, 126, 232.

Medicine, Reports on—Dr. J. R. Charles, 54; Dr. R. Shingleton Smith, 108.
Menthol, Pharmacology of, 70.
Military medicine in England, History of—Dr. G. Parker, 129.
Moore, C. A.—Report on surgery, 58.

Nervous cretinism, 62.
Neuritis and its successful treatment—N. Davies, 81.
Nixon, Dr. J. A.—Cotton-seed dermatitis and its cause, pediculoides ventricosus, 73.
Norgate and Dr. I. Walker Hall, R. H.—An outbreak of institutional dysentery due to the Y bacillus, 44.

Obituary, 123, 228.
Oxygène, Drainage continu par le gaz—Dr. Renoirdre, 39.

Parker, Dr. G.—The foundation of the Medico-Chirurgical Society, and the history of military medicine in England, 129.
Pediculoides ventricosus, cause of cotton-seed dermatitis—Dr. J. A. Nixon, 73.
Per-nasal operation for frontal sinus suppuration, On the—Dr. P. Watson-Williams, 24.
Polyneuritis of the motor type, On toxic—Dr. J. M. Clarke, 9.
Poverty and tuberculosis, 112.

Renoirdre, Dr.—Drainage continu par le gaz oxygène, 39.
Royal mineral water hospital, Patients treated, 126.

Sanatorium work in Bristol, 127.
Sarcoma of the long bones, 58.
Second southern general hospital, Changes in staff, 218.
Sepsis and antisepsis in wounds, 206.
Sheppard, E. J., Obituary notice of, 231.
Shingleton Smith, Captain F., Obituary notice of, 229.
Shingleton Smith, Dr. R.—Report on medicine, 108.
Short, A. R.—Report on surgery, 206.
Sickness insurance for medical men, 225.
Sight, The evolution of the sense of —F. R. Cross, 163.
Society, Bristol Medico-Chirurgical, 71, 226.
Stack, E. H. E.—Gunshot injuries to the eyes, 198.
Stasis, Intestinal—Sir W. A. Lane, 1.
Status lymphaticus, 63.
Stools, Clinical examination of—Dr. G. H. H. Almond, 95.
Surgery, Reports on—C. A. Moore, 58; A. R. Short, 206.
Sympathetic nervous system and the gastro-enteric functions, 55.

Therapeutic notes, 68.
Tuberculosis campaign, Notes on, 108.
Tuberculosis officer, Duties of, 109.

University of Bristol, Examination successes, 72, 231.

Warfare, Cardiac disorders in—Dr. C. Coombs, 149.
Watson-Williams, Dr. P.—On the per-nasal operation for frontal sinus suppuration, 24.
Williams, Dr. E. C.—Report on diseases of children, 61.
Women doctors with the army in the field—Dr. Helen B. Hanson, 88.
Wounds, Sepsis and antisepsis in, 206.

